# Socio-Structural Factors and HIV Care Engagement among People Living with HIV during the COVID-19 Pandemic: A Qualitative Study in the United States

**DOI:** 10.3390/tropicalmed7100259

**Published:** 2022-09-23

**Authors:** Jacob Bleasdale, Lucia A. Leone, Gene D. Morse, Yu Liu, Shelby Taylor, Sarahmona M. Przybyla

**Affiliations:** 1 Department of Community Health and Health Behavior, School of Public Health and Health Professions, University at Buffalo, Buffalo, NY 14214, USA; 2 Center for Integrated Global Biomedical Sciences, Department of Pharmacy Practice, School of Pharmacy and Pharmaceutical Sciences, University at Buffalo, Buffalo, NY 14214, USA; 3 Department of Public Health Sciences, School of Medicine and Dentistry, University of Rochester, Rochester, NY 14642, USA

**Keywords:** people living with HIV, social determinants of health, HIV care continuum, qualitative methodology, food insecurity, COVID-19 pandemic

## Abstract

Achieving HIV prevention goals will require successful engagement in each stage of the HIV continuum. The present study sought to understand the ways in which socio-structural factors influence HIV care engagement among people living with HIV (PLH) within the context of the ongoing COVID-19 pandemic. Twenty-five PLH were recruited from January to October 2021. Semi-structured interviews discussed various socio-contextual factors that influenced engagement in HIV-related care as a result of the pandemic. A thematic content analysis reported semantic level themes describing factors influencing HIV care following an integrated inductive–deductive approach. Qualitative analysis revealed three themes that either supported or hindered engagement in care within the context of the COVID-19 pandemic: (1) social determinants of health, (2) social support, and (3) modes of healthcare delivery. The results underscore the need to assess socio-structural factors of health as means to promote successful engagement in the HIV care continuum and shed new insights to guide future practice in the era of COVID-19.

## 1. Introduction

Engagement across the HIV care continuum, which includes diagnosis, linkage to care, receipt of care, retention in care, and achievement and maintenance of viral suppression, is vital for managing HIV in the United States (U.S.) [1,2]. Despite advancements in both treatment and prevention, data indicate low rates of engagement in nearly every step of the continuum. While 74% of PLH had received some care in 2020, only 51% were retained in care, and approximately 65% had successfully attained viral suppression [2].

Attaining viral suppression is essential to Treatment as Prevention (TaSP) efforts which are a critical tenant of U.S. Ending the HIV Epidemic (EHE) and Undetectable Equals Untransmittable (U = U) initiatives [3]. While TaSP emphasizes the last stage of the continuum (i.e., viral suppression), engagement in the preceding steps is vital. Timely receipt of care after diagnosis has been associated with lower viral loads [4], reduced HIV transmission [5], and delayed progression to Acquired Immunodeficiency Syndrome (AIDS) [6]. Further, sustained retention in care is vital for successful HIV treatment. Decreased retention is associated with reduced antiretroviral therapy (ART) adherence [5], increased risk of viral resistance [7], and HIV-related mortality [8].

Despite efforts to engage PLH in care, multiple social and structural determinants impede successful engagement. The effects of social determinants on HIV care are widely recognized [9]. Recent literature has illustrated strong, population-level associations between social determinants, risk of HIV infection, and disease progression [10,11]. Socioeconomic inequities, such as housing instability [12], income insecurity [13], transportation barriers [13], and food insecurity [14,15] have been associated with decreased care engagement and poor clinical outcomes.

The effects of the COVID-19 pandemic have exacerbated these concerns [16,17,18] and have resulted in healthcare disruptions for PLH [19]. Research during the first twelve months of the pandemic identified impacts on finances [20,21,22], food insecurity [21,23], and access to healthcare services [20,22,23] among PLH. A cross-sectional survey conducted from April–May 2020 illustrated greater financial stress and decreased access to care among PLH [20]. Another study among PLH found approximately 40% lacked access to food because of the COVID-19 pandemic, and nearly 20% missed a scheduled HIV care appointment within the past month [23]. Research has also indicated PLH may be at heightened risk for incomplete viral suppression given the pandemic’s impact on various social and structural factors that inhibit engagement in care [24]. Yet, few qualitative studies have examined how the pandemic has impacted these social and structural determinants and their resulting effects on HIV care [21,22,25]. For instance, a rapid qualitative assessment among PLH illustrated the impact of COVID-19 on various social determinants, including employment and social support, but did not assess how these impacts further influenced HIV care engagement [22].

The influence of the COVID-19 pandemic on pre-existing health inequities among PLH has the potential to have long-lasting effects on individual and public health [24]. While there is a growing body of research examining the effects of the COVID-19 pandemic on HIV care [16,17,18], the majority of work was performed during the early months of the pandemic. To date, there is a lack of research capturing the continued influence of the pandemic on various socio-structural factors that have historically hindered care engagement for PLH. As the U.S. continues to manage COVID-19 outbreaks, such explorations are critical for understanding the additional challenges PLH face to successful care engagement within the era of COVID-19. Given the lack of qualitative research in this area, the primary aim of this study was to explore the ways in which socio-structural factors influenced care engagement among PLH during the COVID-19 pandemic.

## 2. Materials and Methods

### 2.1. Participants and Procedures

Participants were eligible if they were: (1) 18 years of age or older, (2) English speaking, (3) living with HIV, (4) currently prescribed ART for at least 30 days, and (5) currently receiving or had received food assistance in the past 30 days. Food assistance was defined as receiving Supplemental Nutrition Assistance Program (SNAP) benefits or visiting a food pantry in the past 30 days. Participant recruitment occurred between January–October 2021 with a combination of passive and active approaches. Flyers were distributed to sexual health clinics in Western New York (WNY) and the WNY Lesbian, Gay, Bisexual, Transgender, and Queer (LGBTQ+) Pride Center. Participants were also actively recruited from a health service organization with a large presence in the WNY LGBTQ+ community. Interested persons were screened for eligibility and completed a demographic survey. Eligible participants were provided with a study information sheet and were scheduled for an interview.

Interviews were conducted via phone, Zoom, or FaceTime, depending on participant preference. Participants first completed an interviewer-administered survey assessing various aspects of their health, including food security status (Household Food Insecurity Access Scale) [26], HIV-related characteristics (e.g., year of HIV diagnosis, viral suppression status) and ART adherence (Visual Analog Scale) [27].

A semi-structured interview guide then directed discussion about factors that influenced care engagement during the COVID-19 pandemic. Prior to data collection, the semi-structured interview guide was reviewed by experts in HIV prevention, HIV care, and food access, which included community health workers, pharmacists, and public health practitioners and researchers. Expert reviewers provided feedback pertaining to the nature of the interview questions, which were adapted accordingly. Interviews continued until data saturation was achieved. All participants received a $40 Amazon electronic gift card. The University at Buffalo Institutional Review Board approved all study procedures with a waiver for written informed consent.

### 2.2. Data Analytic Plan

Interviews were audio-recorded, transcribed verbatim, and ranged in length from 23 to 53 min (*M* = 34.9 min, *SD* = 9). We conducted a thematic content analysis to report semantic level themes describing HIV care engagement following an integrated inductive-deductive approach [28]. This approach allowed the researchers to develop and organize the codebook based on previous literature and allowed for the generation of new codes as well. The analytic process occurred in five phases. In phase one, three researchers with expertise with qualitative data analysis (one Research Assistant (RA), one Assistant Professor and the Principal Investigator (PI)) familiarized themselves with the data and generated an initial codebook based on previous literature. In phase two, the PI and RA reviewed a subset of transcripts (n = 5) to generate additional codes. The PI and RA met weekly to discuss any discrepancies and revise the codebook. In phase three, the PI and RA recoded the original five transcripts using the revised codebook to ensure consistency. In phase four, the PI and RA independently coded the remaining subset of transcripts (n = 20) and updated the codebook when new codes emerged. In the final phase, we conducted a cross analysis to identify themes by focusing on codes related to social determinants, care engagement, and COVID-19 impacts. The researchers discussed all themes and selected representative quotes for each.

We assessed the rigor and trustworthiness of the data using criteria set forth by Guba (1981) [29]. Credibility was assessed through the PI’s assessment of the interview guide prior to each data collection session. Post-interview assessments were conducted to appraise the authenticity of responses. To ensure dependability and confirmability, the PI adhered to all study protocols and detailed documentation occurred throughout the entire data collection process. We upheld these criteria during the analytic phase, given the researchers met on a weekly basis to discuss the accuracy of codes, reconcile any discrepancies, and reach consensus.

## 3. Results

### 3.1. Participant Characteristics

Of the 25 participants, the majority self-identified as male (52%), Black/African American (68%), and straight/heterosexual (60%). Ages ranged from 24 to 85 years (*M* = 40.4 years, *SD* = 17.9). Self-reported past-month ART adherence ranged from 75% to 100% (*M* = 95.5%, *SD* = 6.0), corresponding to optimal adherence (>95%); the majority self-reported viral suppression (64%). More than half of participants were food insecure (56%). Additional participant characteristics are presented in Table 1.

Thematic analysis revealed three themes that supported or hindered care engagement in the context of the ongoing COVID-19 pandemic: (1) social determinants of health, (2) social support, (3) modes of healthcare delivery. Table 2 presents the frequency and number of participants who endorsed each theme and sub-theme.

### 3.2. Social Determinants of Health

The COVID-19 pandemic impacted various determinants of health which negatively influenced HIV care engagement. Specifically, unstable income, inadequate housing, and food insecurity negatively affected medication adherence and HIV clinic attendance.

#### 3.2.1. Unstable Income

Most participants (n = 16) discussed how economic stressors, such as being unemployed as a result of the COVID-19 pandemic, limited their care engagement. As one participant stated, “I lost my job because of COVID-19 and my income has been really reduced… having problems with my finances makes it difficult for me to be able to get the care I need for my HIV” (35-year-old Black female). Other participants described how their income instability took precedent over their care. The pandemic heightened participants’ concerns about affording everyday necessities, which made attending their HIV appointments less of a priority. As described by one participant, “Currently I’m not working. So, sometimes I don’t have money. When I’m worrying about my next paycheck and where I’ll get money, the last thing on my mind is my doctor visits” (24-year-old Black female).

Participants described how unstable income as a result of the COVID-19 pandemic elicited mental health concerns. Feelings of fear, worry, and anxiety arising from a lack of income influenced poor care engagement and medication adherence. As described by one participant, “I don’t have a job, so I keep worrying about money, so I end up forgetting to take my medication” (24-year-old Black male).

#### 3.2.2. Inadequate Housing

Although more than two-thirds of participants reported stable housing, participants descried how the pandemic contributed to inadequate and unsafe housing. This was a prevalent factor influencing ART adherence for most participants (n = 14). Participants discussed how unstable or poor housing negatively affected their willingness and ability to take their medication. One participant stated, “My current living situation is quite poor because of the COVID-19 pandemic [and] it makes it hard for me to take my medication” (24-year-old Black male). At times, lack of housing influenced prolonged treatment interruptions. As described by one participant:

When you feel like you have no stable roof over your head, what’s the point? You can’t keep track of all your medicine when you’re moving around, you just can’t. And there’s no way you can continue being adherent when you got to worry about where the hell you’re going to live, because that’s the last thing on my mind (55-year-old Black male).

#### 3.2.3. Food Insecurity

Participants (n = 14) discussed experiencing food insecurity as a consequence of the COVID-19 pandemic. As described by one participant, “With the pandemic, I lost my job, and my family was also not working. We had to start limiting the food we were eating and start eating around two meals a day in order to have enough for tomorrow” (25-year-old Black male). Periods of food insecurity led to the erosion of mental wellbeing, which impacted medication adherence and treatment engagement. Feelings of anxiety, depression, and worry about not knowing where their next meal would come from often detracted participants from taking their HIV medication. One participant stated:

Sometimes I’m not so sure about where my next meal will come from and I’m not sure if I will get a balanced diet…it slowly puts me into a depressed state and that makes it hard for me to take my medicine and care for myself (25-year-old Black female).

During the pandemic, attaining food often took precedence over taking HIV medication. As described by one participant, “When you’re going to the supermarket and you see stuff and you can’t afford to buy it, the last thing on my mind is taking my HIV medication. I’m hungry. I don’t want to take my [HIV] medication” (55-year-old Black male). Participants also discussed the physical effects of food insecurity. Feeling weak, slow, and sluggish were commonly reported, often the result of lacking food. Many participants described how these feelings led to disruptions in care, reduced their motivation to take their ART medication, and discouraged HIV clinic attendance. As one participant described, “Physically, I didn’t have the motivation and the strength to take my medicine or go see my doctor because I was worried about going without food” (27-year-old White male).

### 3.3. Social Support

Friends, family, social service providers, and clinicians provided participants with emotional, instrumental, and informational support during the COVID-19 pandemic, which promoted care engagement.

#### 3.3.1. Emotional and Instrumental Support from Family and Friends

Most participants (n = 22) described receiving emotional and instrumental support from family and friends during the pandemic. Emotional support motivated participants to remain adherent to their medications. When asked what support she had during the pandemic to help her take her HIV medication, one participant stated, “My family and kids. My family is always there for me. They always say, ‘you could live, just take your medicine and you will live’” (58-year-old Black male).

Instrumental support from family and friends promoted care engagement. Participants recounted instances where loved ones provided financial and transportation support during times when the COVID-19 pandemic had impeded such needs. This necessitated medication adherence and appointment attendance. One participant stated:

I receive support from my relatives and friends. They have been so supportive of me. Like when I moved in with one of my relatives, he prayed for me, bought me food, and he even was giving [me] bus fare to go my doctors’ appointments and get my HIV medicine. I manage my HIV through the support I receive from friends and family (27-year-old White male).

#### 3.3.2. Emotional and Informational Support from Clinicians and Social Service Providers

Participants (n = 21) also described receiving emotional and informational support from clinicians, case managers, care coordinators, and counselors. Clinicians encouraged participants to remain adherent to their medication and keep up with their appointments. Participants often embraced this support, as described by one participant, “I feel like my doctor is a supportive pillar. She’s very encouraging. She’s not judgmental. That motivates me to see her” (35-year-old Black female).

At times, participants recounted how the COVID-19 pandemic made it difficult to receive transportation, housing, and food, which impeded care. Clinicians and social service providers offered participants informational support to meet their everyday needs. This assistance supported care engagement. As described by one participant, “I have case managers that call and are always checking up on me, telling me what I am supposed to be taking every day, what appointments I have, if I need anything. That helps me be adherent” (44-year-old Black male).

### 3.4. Modes of Healthcare Delivery

Participants described how the ongoing COVID-19 pandemic influenced various modes of healthcare delivery. These concerns commonly arose from fears and worries about contracting SARS-CoV-2 and transitions to telehealth services.

#### 3.4.1. Concerns about Contracting SARS-CoV-2

Most participants (n = 13) described how the COVID-19 pandemic led to care disruptions. Participants described skipping their in-person HIV appointments to mitigate potential exposure to SARS-CoV-2. As described by one participant, “I skip my doctor’s appointments because I don’t want to get COVID-19” (25-year-old Black female). Another participant echoed a similar sentiment, “I am not going to my appointments because I don’t want to go where people are sick with COVID-19” (40-year-old White male).

The pandemic also influenced participants’ medication adherence, mostly due to delays in receiving them in-person from pharmacies and clinics. When asked how the pandemic influenced their medication adherence, one participant said, “When the pandemic happened, I fear[ed] contracting the virus, [so] I stopped going to the pharmacy to get my medication…so at times, I had no medication to take” (27-year-old White male).

Concerns surrounding COVID-19 were not only motivated by contracting the virus, but by its potential effects on people with pre-existing medical conditions, such as HIV. This fear prevented participants from attending their clinic appointments. This is evident in one participant’s statement:

I missed a couple appointments due to COVID-19 because I was worried. I already have HIV, so I am afraid of getting another virus. I missed several visits because I didn’t want to go there and get another illness on top of the HIV (24-year-old Black female).

#### 3.4.2. Transitions to Telehealth

Nearly all participants (n = 23) had experiences receiving HIV care via telehealth during the pandemic. These experiences were mixed, as participants often recounted both positive and negative sentiments. Some participants viewed telehealth positively because it allowed for flexibility. As one participant described, “I can do them [virtual appointments] from anywhere. Even if I’m at work, I can just go somewhere private and have a conversation with my doctor. Things are much easier virtually” (25-year-old Black male).

Telehealth was also preferred among participants who faced barriers to in-person appointments, such as income and transportation. As one participant stated, “With virtual appointments, they have saved me from the burden of transportation costs because there was a time when I didn’t have proper employment and I didn’t have any money to take the bus [to the clinic]” (27-year-old White male). Others felt more comfortable with telehealth because it provided more time and privacy. As one participant described, “I’m more comfortable talking to my doctor because it’s a more private conversation with him, unlike when I go to the office” (26-year-old White male).

The most commonly cited challenges to telehealth were connectivity issues and concerns regarding quality of care. At times, poor internet connections caused participants to miss their appointments. As one participant said, “Sometimes I have a network failure and I am not able to meet with him [doctor] and I miss my appointment” (27-year-old White male). Others described issues with the telehealth software. As described by one participant, “Oh, I hate it. It’s terrible. The system they use is terrible and it only works half of the time” (32-year-old Black male).

Some participants also expressed concerns regarding the quality of their telehealth appointments. Participants believed the level of care they required was not obtainable through virtual meetings with their clinician. As one participant stated, “I hate it [telehealth]. I like in-person [appointments] because they are able to do everything, like, weigh you, talk to you, draw your blood” (32-year-old Black male). Others felt that telehealth limited patient-provider interactions. As described by one participant:

I don’t want to do telehealth. I want to be in his face because I want to see what’s going on. I want to be in-person because it’s my health at the end of the day. I’m not going to keep myself from going to the doctor’s office and do telehealth, it just doesn’t work that way for me (55-year-old Black male).

## 4. Discussion

Current federal HIV initiatives seek to increase the proportion of PLH who are linked to care within one month of diagnosis to 85% and increase the proportion of PLH who are virally suppressed to 80% [2,3]. However, data illustrate only 80% of PLH were linked to care within one month of diagnosis and only 65% of PLH had attained viral suppression in 2020 [2]. The COVID-19 pandemic has likely impacted federal HIV goals due to its influence on various social and structural drivers that impede successful engagement in care. This study illustrated how the ongoing COVID-19 pandemic influenced various socio-structural factors that either hindered or promoted care engagement among PLH.

Our results support findings from previous studies that have demonstrated the impact of COVID-19 on income [20,21,22]. Participants in our study described periods of income instability as a result of the COVID-19 pandemic. Similar to previous work, our participants described losing their jobs and were unable to find additional work because of the pandemic [22]. Our results extend the current work to illustrate how these experiences either exacerbated or initiated care disruptions. For many participants, paying for housing, food, and other necessities took precedent over affording means to attend their doctor’s appointments and medication co-pays.

Most participants described how the pandemic impacted their housing, which further influenced their HIV care. Participants often discussed periods of time where securing safe and stable housing took priority over taking their medication. This challenge frequently became a source of worry, which at times, necessitated medication non-adherence. While previous work has illustrated the impact of COVID-19 on housing instability among PLH [20,30], our study is among the first to qualitatively examine the ways in which COVID-19, housing status, and medication adherence operate. Current literature has illustrated increased stress and anxiety among PLH during the pandemic [16]. As stress is correlated with medication adherence among PLH [31], it is likely that pandemic-related housing instability impacts medication adherence via stress pathways. In addition, our results are not fully consistent with a recent meta-analysis examining the effects of housing instability on medication adherence [32]. Harris and colleagues (2017) found that the risk of medication nonadherence was higher among homeless PLH or PLH living in transient housing compared to PLH living in stable housing; however, the effect size was small. It is possible that our diverging results illustrate the nuanced and multi-faceted effects of the COVID-19 pandemic on housing instability among PLH. Further, given the lack of standard measures for both housing status [33] and medication adherence [34], it is likely that the nuanced relationship between the two phenomena are not accurately captured via quantitative measures.

Recent work has illustrated that food insecurity is associated with poor HIV outcomes via nutritional, behavioral, and mental health pathways [35]. Such concerns have been exacerbated by the COVID-19 pandemic [21,23,36,37]. Our study echoed similar results, as participants described how food insecurity influenced medication adherence and appointment attendance via mental and physical health pathways. Feelings of worry and depression, because of food insecurity, negatively influenced participants’ motivation to take their medication [38,39]. Further, participants described the impacts of food insecurity on attending their medical appointments, primarily through physical health pathways. Participants missed scheduled appointments due to feeling weak and sluggish, which was often the result of prolonged periods of food insecurity. Consistent with Gwadz and colleagues (2021), our results suggest that current support benefits to alleviate food insecurity, such as SNAP, may not adequately address food-related needs during times of crises, such as the COVID-19 pandemic [21].

Despite current research illustrating the effects of COVID-19 on social isolation among PLH [16,22,40], participants in our study described how the pandemic had little influence on their ability to receive support from friends, family, clinicians, and social service providers. Our results highlighted the importance of social support for successful care engagement during the pandemic. Similar to previous work [21], participants described receiving emotional and instrumental support from family and friends. Our study found that social support from loved ones provided participants with intrinsic motivation to remain adherent to their medications and clinic appointments. While previous work noted that healthcare providers offered emotional support for PLH during the pandemic [21], our results illustrated how social services providers and clinicians provided informational support as well. This combination of emotional and informational support often became internalized as intrinsic motivation for participants to adhere to their medical care [41,42]. In addition, participants also received resources from providers to obtain everyday necessities that were impacted by the COVID-19 pandemic, such as food and transportation. This finding highlights the importance of establishing strong patient-provider relationships beyond a siloed approach to address only medical needs and is consistent with previous research demonstrating the positive role of social support on the overall health and quality of life of PLH [41,42].

Participants discussed how the COVID-19 pandemic influenced their ability and willingness to receive HIV care. Consistent with previous work [18,21,22,23,43], participants discussed how fears of infection and social distancing guidelines necessitated missed appointments and hindered medication adherence. In an effort to adhere to public health social distancing guidelines during the pandemic, Ryan White Programs encouraged the use of telehealth to allow PLH to receive their medical care without having to travel to clinics [44]. As described in our study, experiences with telehealth were mixed. Participants described internet connectivity and inadequate quality of care as barriers. Similar to Galaviz and colleagues (2022), our participants were concerned that vital components of their care, such as drawing blood and having viral loads measured, would not be met through telehealth appointments [45]. Despite such concerns, participants also noted many benefits, such as privacy and reduced transportation barriers [25,46,47]. Most importantly, telehealth provided participants with the autonomy to engage in care at a convenient time and location.

Our qualitative results suggest the need to amplify public health strategies that increase HIV care engagement and retention, particularly as a result of the ongoing COVID-19 pandemic. Strategies at the individual and structural levels have the potential to increase engagement and retention in care [1,48]. One strategy is Data-to-Care (D2C), a public health approach that uses HIV surveillance to support engagement in the care continuum [49]. One tenant of D2C is to identify PLH with detectable viral loads who are engaged in care and work with their healthcare provider to attain viral suppression [49]. In particular, step 3 of D2C indicates the use of Health Department and Healthcare Provider Models to facilitate linkage and engagement to outreach services (e.g., case management) [49]. Initial research has demonstrated the success of D2C programs for increasing care engagement among PLH, particularly among those who lacked recent HIV-related laboratory testing [50]. Yet, research among key stakeholders has highlighted the need for D2C programs to address barriers such as transportation and housing [51]. Our results highlight the need to integrate resources that address material needs, such as income, housing, and food, within D2C programs to facilitate successful engagement in the care continuum.

### Limitations

This study should be interpreted within the context of its limitations. First, while our sample was demographically reflective of PLH in the U.S. (e.g., male, Black/African American) [2], our sample was less diverse with regard to geography and sexual orientation. Second, data analyzed for our study used semi-structured interviews to facilitate discussions, which may present social desirability bias. Third, this study is limited by recall bias, as health-related measures (e.g., medication adherence, viral suppression) were self-reported and not confirmed with medical records. Despite these limitations, our study offers valuable insights on the impact of various socio-structural factors on HIV care within the context of the ongoing COVID-19 pandemic.

## 5. Conclusions

This study explored the ways in which socio-structural factors influenced care engagement among PLH during the COVID-19 pandemic. Findings from this study highlight the impact of the ongoing COVID-19 pandemic on HIV care engagement through its influence on various factors. Our results underscore the need for future research that employs both qualitative and quantitative methods to prospectively study the immediate and long-term effects of the COVID-19 pandemic on care engagement for PLH. Such work can inform the development of novel interventions that will allow health systems to approach HIV care with a public health approach that emphasizes the social, structural, and medical needs of PLH, particularly in light of the continually changing contexts of the COVID-19 pandemic.

## Figures and Tables

**Table 1:** Participant Characteristics (N = 25).

Characteristic	n (%)
**Age [M (SD)]**	40.4 (17.9)
**Sex**	
Female	12 (48.0)
Male	13 (52.0)
**Race**	
Black	17 (68.0)
White	7 (28.0)
Other (mixed)	1 (4.0)
**Ethnicity **	
Hispanic/Latino(a)	2 (8.0)
**Sexual Orientation**	
Straight	15 (60.0)
Gay	5 (20.0)
Bisexual	5 (20.0)
**Education Level**	
High school or Lower	8 (32.0)
Some College or Higher	17 (68.0)
**Employment Status**	
Employed	5 (20.0)
Unemployed	15 (80.0)
Income level *	
<$20,000	16 (60.0)
$20,000–$40,000	5 (20.0)
>$40,000	3 (12.0)
**Housing Status **	
Stable	17 (68.0)
Unstable	8 (32.0)
**Food Security **	
Food Secure	11 (44.0)
Mildly Food Insecure	1 (4.0)
Moderately Food Insecure	6 (24.0)
Severely Food Insecure	7 (28.0)
**Years since HIV diagnosis [M (SD)]**	13.2 (11.3)
**Percent Past 30-day ART Adherence [M (SD)]**	95.5 (6.0)
**Virally Suppressed**	16 (64.0)

Note. * Total does not sum to 100% due to missing data.

**Table 2:** Counts and Frequency of Themes.

Primary Theme	Secondary Theme	Number of Excerpts	Number of Participants (N = 25)
Social determinants of health	Unstable income	25	16
	Inadequate housing	21	14
	Food insecurity	26	14
Social support	Emotional and instrumental support from family and friends	65	22
	Emotional and informational support from clinicians and social service providers	58	21
Modes of healthcare delivery	Concerns about contracting SARS-CoV-2	23	13
	Transitions to telehealth	60	23

## Data Availability

The interview guide is available from the corresponding author upon reasonable request.
